# Multipotent to Pluripotent Properties of Adult Stem Cells

**DOI:** 10.1155/2013/813780

**Published:** 2013-12-15

**Authors:** Deepa Bhartiya, Kenneth R. Boheler, Pranela Rameshwar

**Affiliations:** ^1^Stem Cell Biology Department, National Institute for Research in Reproductive Health, Mumbai 400 012, India; ^2^Stem Cell and Regenerative Medicine Consortium, University of Hong Kong, Hong Kong; ^3^Rutgers University School of Biomedical Health Sciences, NJ 07103, USA

Stem cells have captured the attention of both researchers and the public alike because of the promise of tissue regeneration, drug screening, and organogenesis. Stem cells are broadly classified as embryonic or adult with differentiation capacities ranging from pluripotent and multipotent to unipotent. In general, pluripotent stem cells are ascribed to cells derived from the inner cell mass of blastocyst stage embryos or to those generated experimentally using reprogramming factors. Adult stem or progenitor cells are generally tissue-restricted but reside in most organs. While the use of pluripotent stem cells has been limited to a few clinical trials due to safety concerns, adult stem cells have shown evidence of safety and are broadly employed in both clinical trials (http://www.clinicaltrial.gov/) and clinical practice. Transplantation of adult bone marrow hematopoietic stem cells, for example, represents a standard method of clinical care for autoimmune diseases and hematological disorders. While hematopoietic stem cells cannot be expanded *in vitro*, other stem cells such as mesenchymal stem cells (MSCs) can be easily expanded from autologous and allogeneic sources for clinical testing. Moreover, adult-derived testicular spermatogonial stem cells (SSCs) have been reported to be the only cells in the body that can be dedifferentiated/reprogrammed to a pluripotent state *in vitro* and grown into ES-like colonies.

It has been advocated that adult stem cells have remarkable plasticity, transdetermination, and transdifferentiation ability. These properties have raised hope that adult stem cells could become a universal source of stem cells for tissue/organ repair in lieu of embryonic stem cells, which can easily form tumors. MSCs, for example, can be isolated from various tissues like bone marrow, gut, lungs, liver, blood, adipose tissue, umbilical cord Wharton's jelly, dental pulp, amniotic fluid, and so forth and have the ability to form bone, cartilage, adipose tissue, skeletal muscle, liver, neurons, skin, pancreatic islets, endothelial cells, intestine, renal, epithelial, and germ cells. More importantly, MSCs can be given across allogeneic barriers. Nevertheless, many trials with MSCs have shown only marginal benefits and some have proposed that the effects may be more paracrine in nature than being regenerative.

This special issue, comprised of 10 articles, focuses on adult stem cells that examine the following claims: Why are MSCs ubiquitous? Are they indeed stem cells or just stromal cells which constitute the somatic niche for tissue specific stem cells in various body organs? Have we looked carefully at MSCs growing in a culture dish? Are ES-like colonies observed only from testicular biopsy but also from ovarian and endometrial biopsies? The article by D. Bhartiya addresses these issues and reviews the available literature on the presence of novel pluripotent very small ES-like stem cells (VSELs) as a subgroup among MSCs. It also highlights that Oct-4A transcripts need to be studied to conclude pluripotent state rather than Oct-4. An article by Dr. P. Rameshwar's group studied the safety issue of MSCs since these stem cells have been shown to support tumor growth. Other papers have discussed various sources of adult stem cells including dental pulp stem cells (S. Arrifin et al.), satellite stem cells in skeletal muscles (S. Fujimaki et al.), and bone marrow stem cells (I. Catacchio et al.) and their multipotent properties and transdifferentiation potential. Further characterization of these stem cells and their functional potential is essential. The role of cannabinoid receptor type I in differentiation and survival of MSCs is discussed by A. Gowran et al. M. Akita et al. show that CD133 positive cells have the capacity to form endothelial capillary tubes in 3D culture. Cultures of cardiac explants result in the formation of cardiospheres which contain both stem and progenitor cells implicated in cardiac regeneration (L. Barile et al.). Y. Togo et al. have reviewed the literature on the use of MSCs as a nonviral gene carrier for gene transfer *in vitro. *Finally, N. Sakayori et al. discuss how neural stem/progenitor cells function is regulated by lipids.

The diverse areas of the manuscripts in this issue underscore the wide field of adult stem cell biology. The papers indicate that more in-depth research and functional studies are required to take adult stem cells safely to the clinic for regenerative medicine.

*Deepa Bhartiya* *Deepa Bhartiya* *Kenneth R. Boheler* *Kenneth R. Boheler* *Pranela Rameshwar* *Pranela Rameshwar*

